# The Mediation Role of Relatedness and Competence for Patient Activation: A Longitudinal Study of Older Adults with Chronic Illness

**DOI:** 10.3390/healthcare14121783

**Published:** 2026-06-20

**Authors:** Monica Kaltenbrunner, Maria Flink, Amanda Hellström, Mirjam Ekstedt

**Affiliations:** 1 Faculty of Health and Life Sciences, Linnaeus University, 392 31 Kalmar, Sweden; amanda.hellstrom@lnu.se (A.H.); mirjam.ekstedt@lnu.se (M.E.); 2 Faculty of Health and Occupational Studies, University of Gävle, 801 76 Gävle, Sweden; 3 Department of Neurobiology, Care Sciences and Society, Karolinska Institutet, 171 77 Stockholm, Sweden; maria.flink@ki.se; 4 Stockholm Research and Development Unit for Older Persons, 113 46 Stockholm, Sweden; 5 Academic Primary Health Care—Öland, 391 26 Kalmar, Sweden; 6 Department of Learning, Informatics, Management and Ethics, Karolinska Institutet, 171 77 Stockholm, Sweden

**Keywords:** older adults, patient activation, chronic illness, depressive symptoms, multimorbidity, quality of life, motivational interviewing, mediation, psychological needs, self-rated health

## Abstract

**Background**: Patient activation is associated with both health outcomes and the utilization of healthcare resources. Since various factors influence activation levels among older ill adults, further exploration of this topic is needed. Specifically, we aim to examine the extent to which changes over time in self-rated symptoms of depression are associated with changes in patient activation and to what extent self-rated health status and satisfaction of basic psychological needs (autonomy, relatedness, and competence) have a mediation effect. **Methods**: A longitudinal and correlational design was employed in which two hundred and seven participants with heart failure or chronic obstructive pulmonary disease were recruited from two hospitals in the middle of Sweden. The sample used in this study is the same as that used in a randomized controlled trial. A questionnaire was administered at baseline, and at 30-, 90-, and 180 days post-discharge, involving ratings of depression, patient activation, self-rated health, and satisfaction of basic psychological needs (autonomy, relatedness, and competence). As the results from the original study showed no difference between the two randomized groups in patient activation, the analysis in this study was conducted using a combined sample in which the intervention and control groups were merged. For estimation of the direct effects and the components of indirect effects, we employed multilevel modeling using a linear mixed model, and to test mediation, the stand-alone program RMediation was used. **Results**: Over time, increases in depressive symptoms were associated with reduced patient activation, with this relationship mediated by declines in relatedness and competence. No evidence was found showing that autonomy or self-rated health had a mediation effect. **Conclusions**: The results indicate that older chronically ill individuals may benefit from interventions targeting psychological mediators to improve and sustain activation.

## 1. Introduction

Hospitalization and hospital discharge are potentially associated with harmful events, such as an increased risk for infections and avoidable re-hospitalizations [1,2]. Such events lead to both patient suffering and increased healthcare costs [3,4], highlighting the need to reduce re-hospitalizations. Systematic reviews have identified that the most effective interventions for reducing re-hospitalization seem to be complex interventions [5,6,7] and supporting patients’ ability to be active in the management of their illness [6,7,8,9].

The importance of supporting patients’ ability to engage in self-management has also been emphasized in the chronic care model, highlighting the role of “informed, activated patients” [10,11]. Hibbard et al. (2005) and colleagues [12] have operationalized patient activation as a person’s knowledge, skills, and confidence to manage their own health. An increasing body of evidence indicates that higher levels of patient activation are associated with improved health [13,14,15,16]. Lower levels of patient activation are, in turn, associated with increased use of healthcare resources [14,16,17] and impaired physical and mental health [16,18].

Several interventions have accordingly been developed to improve patients’ levels of activation. A recent systematic review identified 32 patient activation interventions for people with long-term illnesses, of which 18 were randomized controlled trials (RCTs) [19]. Despite several interventions reporting positive results, the meta-analysis showed no significant improvement in patient activation at 6 months [19]. Another meta-analysis of patient activation interventions for people with type 2 diabetes also failed to show a significant improvement in patient activation [20]. Few studies have investigated patient activation in frail older adults. However, studies have found that patient activation levels are low in this group [21,22]. Low levels of patient activation were in Overbeek et al.’s study associated with low health-related quality of life and living in residential care homes [21].

Factors that potentially have an impact on patients’ ability to take an active role in managing their health include disease-related factors such as the complexity and symptom burden of the illness and patient characteristics such as age, functionality and mobility, cognitive capacity, and health literacy [23,24,25]. Growing evidence shows that patient activation is negatively associated with depression [25,26] and positively associated with health-related quality of life (HRQoL), self-efficacy, social support, and health literacy [24,25,27]. The corresponding association, i.e., between low patient activation and decline in HRQoL following hospitalization, has also been found [28]. In addition to depression and HRQoL, there is increasing awareness that activation interventions should include aspects of motivation. The management of illness and healthcare requires not only the ability to take action but also motivation, education, and engagement to persist in these activities [29].

According to the Self-Determination Theory, motivation to engage in activities is related to the frustration or satisfaction of three basic psychological needs: autonomy (acting with volition and interest), competence (feeling capable), and relatedness (feeling connected, understood, and cared for) [30]. People are more likely to be motivated to engage in activities when these needs are satisfied [31].

Even though an association between patient activation, depression, and health-related quality of life (HRQoL) has been established, few studies have explored the longitudinal relationship [24,32]. Calls have been made for longitudinal studies to explore this relationship [24,26,33]. Despite the potential of patient activation interventions, there is still a lack of evidence on how to best support patients to become active self-managers [19,20].

Therefore, this longitudinal and correlational study sought to contribute to a further exploration of how patient activation may be enhanced. In this study, symptoms of depression (PHQ) are conceptualized as a predictor and are hypothesized to influence the outcome variable patient activation (PAM) through the mediators of patients’ self-rated health (EQ-VAS) and their satisfaction with basic psychological needs (NSFS). The aim was thus to examine the extent to which changes over time in self-rated symptoms of depression are associated with changes in patient activation and to what extent self-rated health state and satisfaction of basic psychological needs have a mediation effect.

**Figure 1:**
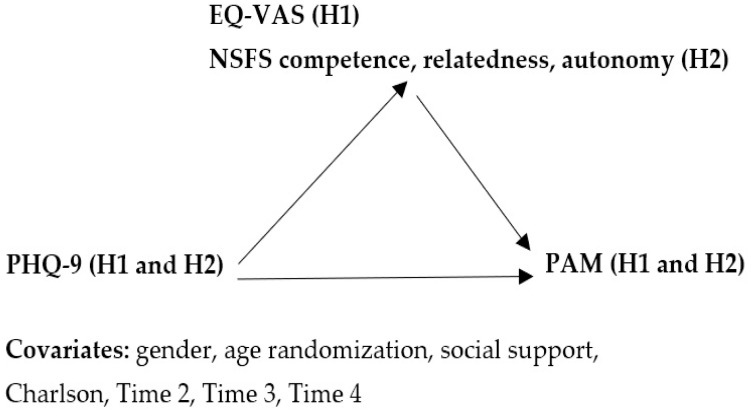
Hypothesized pathways. Model of the two hypothesized pathways between symptoms of depression (PHQ-9), the mediator’s self-rated health state (EQ-VAS) (H1), the satisfaction of basic psychological needs (NSFS) (H2), and the outcome variable patient activation (PAM) and covariates.

Our hypotheses (Figure 1) were as follows:

**H1.**  *Over time, there is a negative association between symptoms of depression and patient activation, and the association is mediated by a decreased self-rated health state.*

**H2.**  *Over time, there is a negative association between symptoms of depression and patient activation, and the association is mediated by decreased satisfaction with basic psychological needs (competence, relatedness, and autonomy).*

## 2. Materials and Methods

### 2.1. Setting and Sample

This longitudinal and correlational study uses data from the Supporting Patient Activation in Transition to Home (sPATH) intervention, which was based on the Self-Determination Theory and motivational interviewing. Participants were randomized to either five post-discharge sessions (n = 103) or a control group (n = 104). The sessions, delivered by a patient activation coach, aimed to support self-management after hospitalization. A comprehensive description of the intervention is available elsewhere [34].

A convenience sample of four healthcare departments in the middle of Sweden was taken. At Karolinska University Hospital, the emergency and pulmonary medicine departments participated, while at Capio St. Goran Hospital, the emergency and cardiology departments participated. In total, these departments had 103 beds.

The inclusion criteria were that the patients had been diagnosed with congestive heart failure or chronic obstructive pulmonary disease, were over 18 years old, and were living in private homes. The exclusion criteria were based on the patients’ prospects to take part in the motivational interviewing sessions: patients with the statement of ‘do-not-resuscitate’ in their medical record, patients who were non-Swedish speaking, and/or patients who had a diagnosis of dementia or mild cognitive impairment were excluded.

Recruitment started in August 2016 and was completed in May 2018. Patients in the four departments who met the inclusion criteria were asked to participate and received written and verbal information about the study from the research staff. Before inclusion, all participants gave their informed consent to participate. No witnesses were involved, but the healthcare professionals always had a say in determining whether the patient was suitable to participate. Written consent to participate was obtained, and the document was stored in a locked cabinet to prevent unauthorized access. Participants were informed that participation consisted of being in an intervention or a control group and that declining to participate did not affect their current or future healthcare. Throughout the data collection and analysis, the authors had access to data that made it possible to identify individual participants.

In total, 207 participants accepted participation. The sample used in this study is the same as that used in a randomized controlled trial [34] (trial registration number NCT02823795), registered on 1 July 2016. While the participants were initially randomized into a motivational interviewing intervention (n = 103) and a control group (n = 104), the original study found no significant difference between these two groups regarding the primary outcome of patient activation [34]. Consequently, the decision was made to pool the groups for the current study. From a statistical perspective, merging the samples maximizes the statistical power required to detect nuanced, indirect longitudinal mediation pathways, thereby reducing the risk of Type II errors. From a theoretical perspective, our primary objective was not to evaluate a specific intervention, but rather to investigate processes that underlie patient activation over time. By pooling the data, we were able to examine these mechanisms across a broader and more diverse sample, enhancing the generalizability and robustness of the longitudinal associations. ‘Randomization’ was included as a covariate in all multilevel models to account for residual group-level variance. For more information about the randomization process and the randomized sample, see Kaltenbrunner et al. [35].

### 2.2. Data Collection

Data was collected using questionnaires at four time points: baseline (T1) and 30 (T2), 90 (T3), and 180 (T4) days after baseline. Two researchers collected baseline data from patients before randomization. Patients who could not respond to the questionnaire in the hospital due to fatigue and/or time constraints related to the discharge were given the option to respond at home. Questionnaires for T2, T3, and T4 were mailed to patients with instructions to respond within one week and return the questionnaire using a postage-paid return envelope. Non-responders received two reminders; before the final reminder, at least one attempt to contact non-responders by telephone or text message was made.

### 2.3. Measures

#### 2.3.1. Patient Health Questionnaire

The independent variable was symptoms of depression. To evaluate this, the nine-item Patient Health Questionnaire (PHQ-9) [36] was used. The PHQ-9 is based on the criteria for depressive disorders in the Diagnostic and Statistical Manual of Mental Disorders, Fourth Edition (DSM-IV). Response alternatives are scored from 0 (not at all) to 3 (nearly every day). The total score is obtained by adding the scores of all items in the questionnaire and ranges from 0 to 27. Scores between 15 and 27 indicate moderate to severe depression. The psychometric properties of the instrument are considered satisfactory [37,38].

#### 2.3.2. Patient Activation Measure

The dependent variable was patient activation and was measured using the 13-item Patient Activation Measure (PAM-13-S) [39]. The instrument assesses patients’ self-reported skills, knowledge, and confidence in self-management concerning their own health and healthcare. Response alternatives are rated on a 4-point Likert scale from strongly disagree to strongly agree, where higher scores indicate higher levels of activation. The Swedish version of PAM-13 was validated by Hellström et al. [40]. The instrument is reliable, although further development has been recommended to improve its validity. Cronbach’s alpha value was 0.81.

#### 2.3.3. Mediators

For the assessment of satisfaction with basic psychological needs, the Need Satisfaction and Frustration Scale (NSFS) was used [41]. The scale consists of three subscales: autonomy, relatedness, and competence, each containing six items. Response alternatives range from never (1) to always (7), and some items need to be reverse-scored before calculating the mean scores for the subscales. Higher scores indicate a higher level of satisfaction with basic psychological needs. The psychometric properties of the Swedish version of the instrument were satisfying [42].

The other mediator was self-reported health and was assessed using EuroQol-visual analog scales (EQ-VAS). EQ-VAS assesses responders’ self-rated health state on the day of assessment, termed as self-reported health. The response is made by marking a point on a vertical scale ranging from 0 (worst imaginable health) to 100 (best imaginable health). The psychometric properties of the instrument are satisfactory [43].

Participants were also asked about gender, age, social support, marital status, and income, and the severity of comorbidity using the age-adjusted Charlson index [44]. The Charlson index was collected only at baseline using healthcare registry data; high scores indicate increased severity of comorbidity.

### 2.4. Statistical Analysis

IBM SPSS Statistics, version 27, was used to describe the participants’ demographic data and to perform multivariate analyses on multilevel and repeated-measures data. The data used in this study were accessed and analyzed between December 2021 and May 2022.

Missing data were missing at random (MAR) and were handled using multiple imputations. A multiple imputation was employed for PHQ-9, EQ-VAS, and NSFS before commencing the multilevel analysis. However, a multiple imputation was deliberately not applied to the PAM items. This decision was made to strictly adhere to the proprietary scoring procedure provided by Insignia Health, which features its own built-in protocol for handling missing data. When using multiple imputations, no cases with missing data are excluded [45]. Replacing missing data using multiple imputations increases the validity and precision of the data and, in turn, enables robust analyses. In this procedure, we chose to generate five datasets, as this has been recommended previously [45]. However, more recently, the suggestion is to create between 20 and 100 imputations [46]. A higher number of generated datasets yields more accurate results, which, in our case, with only five datasets, is a limitation and needs to be considered when interpreting the results.

To test mediating effects (H1 and H2), models involving mediators were created. In both models, PHQ-9 was used as the predictor and PAM as the dependent variable. In the first model, the mediator was EQ-VAS, and the aim was to test whether changes over time in PHQ-9 operated indirectly through EQ-VAS on the dependent variable PAM. In the second model, the mediators were instead the dimensions of autonomy, relatedness, and competence of the NSFS.

For the estimation of direct effects and to retrieve estimates of parameters and their standard errors, which were used to test indirect effects, multilevel modeling was employed using a linear mixed model [47]. To test mediation, estimates of parameters and their standard errors were entered into the stand-alone program RMediation, v 1.2.1, based on R syntax, v 4.5.0 [48]. In the models, random intercepts with repeated measures were used to account for the nested structure of the data within individual participants based on longitudinal data. A compound symmetry covariance structure was employed. Estimation of 95% confidence interval (CI) of the indirect effects was performed using the distribution of the product method in RMediation [48].

Included covariates in the models were gender, age, randomization, social support, the Charlson comorbidity index, and Time. To model the longitudinal trajectories, time was included as a fixed effect, with Time 1 treated as the baseline reference point and Time 2, Time 3, and Time 4 explicitly capturing the subsequent changes from this initial baseline level.

Residuals were inspected visually using histograms and showed no major deviation from a normal distribution. Multicollinearity was assessed using a linear regression analysis, which included all variables in the models. The highest variance inflation factor (VIF) value was 2.17, which indicates no multicollinearity.

Due to study limitations, such as sample attrition, the limited number of imputations, and the fact that the resulting estimates capture a mixture of both between-person differences and within-person changes, the findings should be interpreted with caution.

## 3. Results

**Figure 2:**
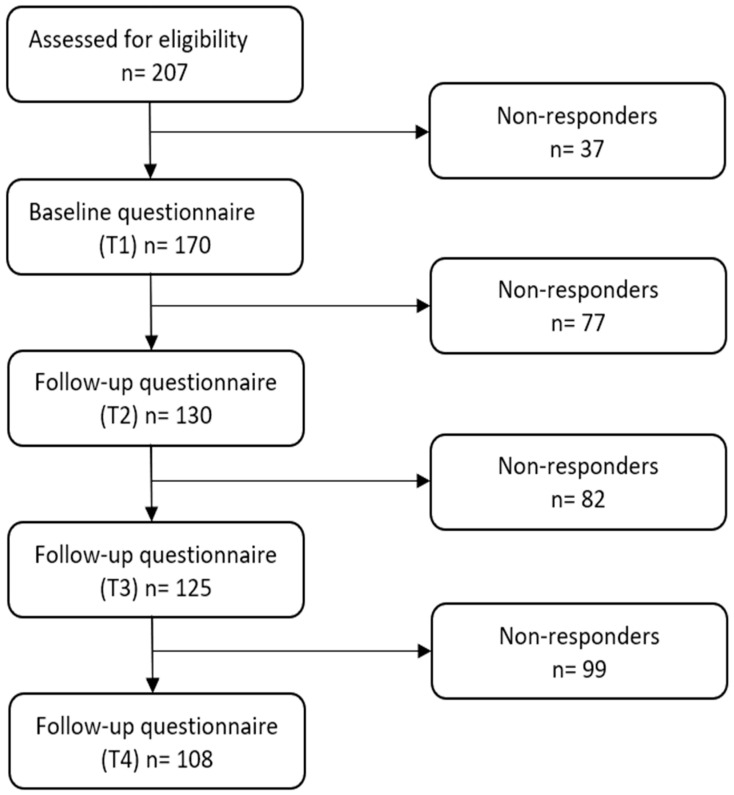
Flowchart of eligible participants. The number of responders at each time point does not add up sequentially because some participants responded intermittently (for example, providing data at baseline and T3, but not at T2 and T4).

Most of the participants were born in Sweden (85%), were men (53%), and the average age was 75 years (SD 10.46). The oldest participant was born in 1923, and the youngest was born in 1977. The mean Charlson index in the sample was 5.94 (SD 2.0). Most participants had a secondary school education (39%), 46% had an income of 10,000–20,000, and 90% perceived that they had social support (see Table 1 for sample characteristics). The response rate decreased during the study, from n = 207 when assessed for eligibility, to n = 108 at T4. Twenty-six participants reported withdrawing due to perceiving confusion, fatigue, or illness. Otherwise, the reasons for dropouts were unknown or because the patient died (Figure 2). In total, 31 individuals were excluded as they did not respond at any time. The attrition rate in this study constitutes a limitation. It is possible that participants with lower baseline patient activation (PAM) or higher depressive symptoms (PHQ-9) were systematically more likely to drop out. Consequently, the remaining sample may suffer from selection bias, meaning the final results might predominantly reflect individuals who were healthier and more highly activated. This potential ‘healthy survivor’ effect must be carefully considered when interpreting the strength and generalizability of the longitudinal findings. An analysis of non-responders (n = 31) showed no statistically significant differences between them and responders regarding gender, age, and Charlson index (*p* = 0.95, *p* = 0.91, and *p* = 0.78).

### Hypotheses 1 and 2: Direct and Indirect Effects of Depression on Patient Activation

**Figure 3:**
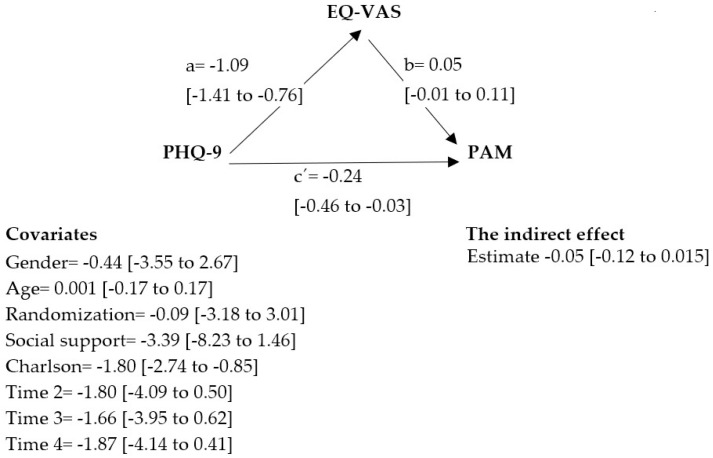
Direct (c′) and indirect effects (estimated by a × b). The predictor symptoms of depression (PHQ-9) and the outcome variable patient activation (PAM), mediated by self-rated health (EQ-VAS). No mediating effect was found for EQ-VAS as the estimate of the indirect effect included zero. The estimate of path a (PHQ-9 to EQ-VAS) and the direct effect, path c′ (PHQ-9 to PAM), did not include zero, meaning that the associations were statistically significant; in these cases, the associations were negative. For path b (EQ-VAS to PAM), the estimate included zero, meaning that the association was not significant. Effects for covariates are shown below the figure. Within the brackets are the 95% confidence intervals for the effect sizes.

H1 was not supported. The indirect effect through EQ-VAS (path a × path b) included zero as it was estimated at −0.05 (95% CI: −0.12 to 0.015). In the model, the direct effect, path c′, was statistically significant and negative (95% CI −0.46 to −0.03) (controlling for the mediator), indicating that higher levels of depressive symptoms are associated with lower patient activation. There was also a significant negative association between symptoms of depression and self-reported health. Path (a) was estimated to be −1.09 (95% CI −1.41 to −0.76), indicating that higher levels of depression are associated with decreased self-reported health (Figure 3).

**Figure 4:**
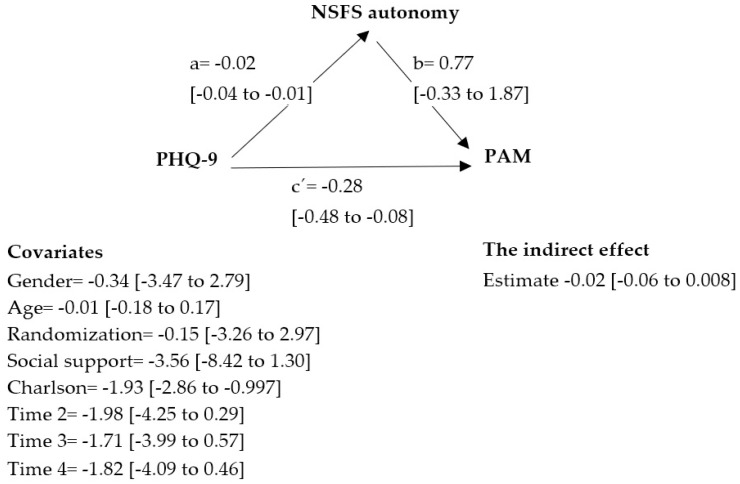
Direct (c′) and indirect effects (estimated by a × b). The predictor symptoms of depression (PHQ-9) and the outcome variable patient activation (PAM) were mediated by the satisfaction of basic psychological needs and autonomy (NSFS autonomy). No mediating effect was found for NSFS autonomy as the estimate of the indirect effect included zero. The estimate of path a (PHQ-9 to NSFS autonomy) and the direct effect, path c′ (PHQ-9 to PAM), did not include zero, meaning that the associations were statistically significant; in these cases, the associations were negative. For path b (NSFS autonomy to PAM), the estimate included zero, meaning that the association was not significant. Effects for covariates are shown below the figure. Within the brackets are the 95% confidence intervals on the effect sizes.

For H2, we hypothesized that, over time, there would be a negative association between symptoms of depression and patient activation, and that this association would be mediated by decreased satisfaction with basic psychological needs (NSFS autonomy, relatedness, and competence). The results for NSFS autonomy showed no mediating effect as the indirect effect (path a × path b) was estimated to be −0.02 (95% CI −0.06 to 0.008) (controlling for the mediator). However, the direct effect, path c′, was significant and was estimated to be −0.28 (95% CI −0.48 to −0.08), showing that higher levels of depressive symptoms were associated with reduced patient activation (Figure 4).

**Figure 5:**
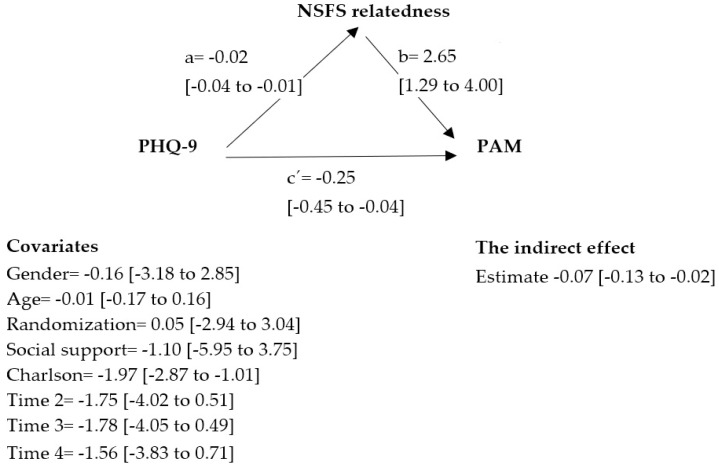
Direct (c′) and indirect effects (estimated by a × b). The predictor symptoms of depression (PHQ-9) and the outcome variable patient activation (PAM), mediated by satisfaction of basic psychological needs and relatedness (NSFS relatedness). A mediating effect was found for NSFS relatedness as the estimate of the indirect effect did not include zero. The estimate of path a (PHQ-9 to NSFS relatedness) and the direct effect, path c′ (PHQ-9 to PAM), did not include zero, meaning that the associations were statistically significant; in these cases, the associations were negative. For the path b (NSFS relatedness to PAM), the estimate did not include zero, meaning that the association was significant. Effects for covariates are shown below the figure. Within the brackets are the 95% confidence intervals on the effect sizes.

For H2, regarding NSFS relatedness (Figure 5), the results showed that changes in symptoms of depression, over time, were statistically significantly associated with changes in patient activation, indirectly through the mediator. For relatedness, the indirect effect was estimated to be −0.07 (95% CI −0.13 to −0.02). The indirect effects were negative, meaning that higher levels of symptoms of depression were associated with lower patient activation, mediated by lower relatedness. The direct effect (controlling for the mediators) was significant for the model including the mediator relatedness with estimates of −0.25 (95% CI −0.45 to −0.04). The results also showed negative associations between symptoms of depression and relatedness. This means that lower levels of symptoms of depression were associated with increased relatedness. Positive associations were found between relatedness and patient activation, meaning that higher patient activation was associated with increased relatedness.

**Figure 6:**
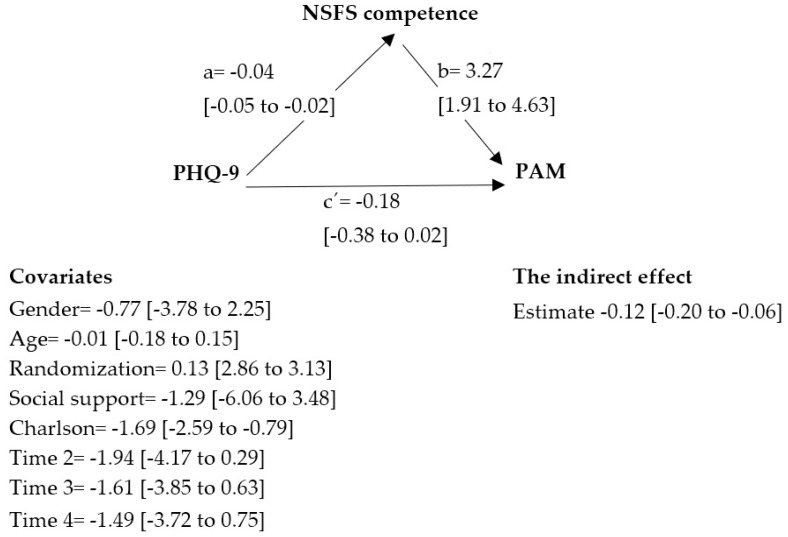
Direct (c′) and indirect effects (estimated by a × b). The predictor symptoms of depression (PHQ-9) and the outcome variable patient activation (PAM) were mediated by the satisfaction of basic psychological needs and competence (NSFS competence). A mediating effect was found for NSFS competence as the estimate of the indirect effect did not include zero. The estimate of path a (PHQ-9 to NSFS competence) and the direct effect, path c′ (PHQ-9 to PAM), did not include zero, meaning that the associations were statistically significant; in these cases, the associations were negative. For the path b (NSFS competence to PAM), the estimate did not include zero, meaning that the association was significant. Effects for covariates are shown below the figure. Within the brackets are the 95% confidence intervals on the effect sizes.

For H2, regarding NSFS competence (Figure 6), the results showed that changes in symptoms of depression over time were statistically significantly associated with changes in patient activation, indirectly through the mediator. For competence, the indirect effect was estimated to be −0.12 (95% CI −0.20 to −0.06). The indirect effects were negative, meaning that higher levels of symptoms of depression were associated with lower patient activation, mediated by lower competence. The direct effect (controlling for the mediators) was non-significant for the model including competence as a mediator, which was estimated as −0.18 (95% CI −0.38 to 0.02). The results also showed negative associations between symptoms of depression and competence. This means that lower levels of symptoms of depression were associated with increased competence. Positive associations were found between competence and patient activation, meaning that higher patient activation was associated with increased competence.

## 4. Discussion

Our results did not support hypothesis 1, as no mediating effect was identified for self-reported health. Our results partially supported hypothesis 2. We observed that an increase in depressive symptoms was associated with decreased patient activation, mediated by diminished relatedness and competence, respectively. No support was found for autonomy as a mediator.

### 4.1. Relatedness and Competence

Relatedness and competence had a mediating effect on patient activation and were not surprising, as motivation to engage in activities is related to relatedness and competence [27]. This aligns with the understanding that depressive symptoms can significantly impact an individual’s perception of being connected, understood, and cared for by others, and their feelings of self-efficacy in accomplishing daily tasks—key components referred to as relatedness and competence within the NSFS [30]. The association between depression and patient activation is well-established [15,18,19,24,25,26,49,50]. However, various factors contribute to depressive symptoms and can subsequently hinder patient activation. Factors associated with depression among older people, as in our sample, include being older than 75 years, having heart disease, diabetes, or stroke, being single, having poor daily physical exercise, and a lack of support [51]. Depressive symptoms themselves are characterized by reduced interest or pleasure, low mood, and diminished self-esteem, which may negatively influence engagement in daily activities [36]. Additionally, late-life depression has been associated with cognitive impairment [43] and challenges in treatment adherence [45]. As these factors may be related to relatedness and competence, they may help explain our finding that reduced need satisfaction in both domains mediated the association between depressive symptoms and patient activation. However, although several factors are relevant for understanding depressive symptoms and patient activation in older populations, this study examined only the mediators EQ-VAS and NSFS.

### 4.2. Autonomy

Autonomy, the ability to make decisions and act according to one’s will [52], is an important factor in promoting patient activation [53]. However, our analysis revealed no mediating effect of autonomy. This outcome could be attributed to various reasons. Autonomy may represent only one of several factors influencing patient activation. Furthermore, in a sample characterized by old age and comorbidity, autonomy alone may be insufficient to mediate the association between depressive symptoms and patient activation. An increased need for care can threaten older people’s autonomy due to their growing dependency on professionals. Nevertheless, autonomy remains a core value in the care of older people, and professionals need to consider how older individuals perceive autonomy in their everyday practice. It is possible that the need for autonomy can be satisfied even if the person is physically impaired. Older individuals in residential care [54] reported that their inner freedom to think and make decisions remained intact even when their functional abilities diminished. This suggests that autonomy does not depend solely on physical capabilities but also on other factors, possibly including cognitive and emotional factors. Participants in our study lived in their private homes, potentially experiencing a high degree of autonomy. Therefore, as our findings indicate, other factors, such as relatedness and competence, or additional influences like social support [55], or health literacy [18,24,25], may play a more substantial role in driving patient activation than autonomy in this context. Moreover, while competence, relatedness, and autonomy are important factors for need satisfaction within the NSFS framework, not all factors have the same impact on the outcome in every study [56], including ours. As earlier studies highlight, there appears to be a need for more tailored and differentiated interventions [53] to meet the diverse expectations and needs of the heterogeneous population of older adults with chronic conditions [57].

### 4.3. Self-Reported Health

Our results showed no mediation effect in the model for self-reported health (EQ-VAS). Earlier studies have reported mixed results concerning these associations. For instance, one study reported that there are different perspectives on the overall health benefits associated with high patient activation [16]. Three studies, a randomized controlled study and two cross-sectional studies, found associations between patient activation and health-related quality of life [24,25]; one study used the single-item EQ-VAS [58]. However, another study found no association between patient activation and EQ-VAS [59]. It is possible that our results may be related to the fact that a single item, the EQ-VAS, was used to assess health. However, according to Macias et al. [60], single items can be as sensitive, valid, and reliable as a scale.

Physical activity has been shown to reduce depression among older adults [61,62] and may also improve patient activation; however, more research is needed in this population [62].

However, engaging in empowering activities may be challenging for older people with comorbidity, as observed in our sample [35]. Tailored interventions, such as health coaching, may therefore help support older patients with long-term conditions, particularly those experiencing symptoms of depression.

### 4.4. Strengths and Limitations

The study presents both strengths and limitations. A limitation is the generalizability of the results, due to our small sample size and the specific population of Swedish older adults with chronic illnesses. The attrition rate is a limitation, and longitudinal research is a challenge, as data often are missing across the longitudinal time points, as in this study. In this study, it is possible that participants with, e.g., the lowest baseline patient activation (PAM) or the highest depressive symptoms (PHQ-9) were more likely to discontinue the study. Consequently, the results may partially reflect a sample of healthier or more resilient individuals. This potential bias should be considered when interpreting the findings.

Another methodological consideration that might be seen as a limitation is the merging of intervention and control groups into a single longitudinal sample. This decision shifts the design from experimental to correlational and can potentially reduce internal validity. Being critical, combining participants who received an active intervention with those who did not could obscure differential group-by-time trajectories or introduce confounding artifactual relationships—essentially masking two distinct group dynamics under a single average. However, we consider our decision to merge the sample to be justified and methodologically sound for the present analysis, particularly because pooling the sample mitigates the risk of an underpowered analysis and ensures more reliable estimates. Given that the primary trial [35] demonstrated an absence of statistically significant or clinically meaningful differences between the arms regarding patient activation, it is possible that the two groups can be considered homogeneous. Furthermore, the original randomization assignment has been strictly controlled as a covariate in all models. Adjusting for randomization and focusing on mechanisms provides insight into mediators of patient activation. Although a sensitivity analysis comparing the intervention and control groups could have been conducted, no such analysis was performed by the group. This decision aligns with the primary aim of the present study, which was to examine pooled longitudinal associations rather than specific intervention effects. Another limitation is the measurement instruments. This includes the use of a single-item measure, such as EQ-VAS, which may limit sensitivity. The Swedish PAM-13 has demonstrated acceptable reliability. However, concerns have been raised because evidence is lacking to conclusively confirm that the instrument represents a single underlying construct [40]. High reliability of an instrument is of paramount importance as it ensures that the measure is stable over time and that the observed changes in scores reflect true variations in patient activation rather than measurement error or random noise. A major advantage of utilizing the instruments we employed is that they are well-known and accepted in international health research. Using a widely established instrument enhances the comparability of our findings with the existing literature, allowing for a broader and more meaningful contextualization of our results within the field of patient-centered care. The lack of a comparison between baseline responders and those who completed the study at Time 4, regarding PHQ-9, PAM, EQ-VAS, and NSFS scores, constitutes a limitation. Without this analysis, it is difficult to rule out a potential attrition bias, where the final sample may disproportionately represent healthier or more resilient participants. This potential bias must be taken into account when interpreting the findings.

As noted earlier, missing data across time points is a common challenge in longitudinal research. The impact of missing data can result in a loss of statistical power and the introduction of selection bias, thereby threatening the validity of the conclusions. A strength in this study was the use of multiple imputations when handling missing data. A major advantage of this approach is that no data are discharged. Furthermore, as noted in the literature, the Rasch-based scoring algorithm of the PAM instrument itself allows for valid score calibration even if individual items are missing within a specific wave, provided that a minimum of 9 out of 13 items are answered.

We chose to generate five imputed data sets as recommended [45]. However, more recently, the suggestion is to create between 20 and 100 imputations [46]. A higher number of generated datasets yields more accurate results, which, in our case, with only five datasets, is a limitation. Furthermore, the longitudinal design with shorter follow-up periods aligns with common practices in the literature [63]. However, basing a study on a sample with these characteristics, as in our study, presents challenges. Recruiting older individuals with severe illnesses for studies, interventions, and treatments is difficult [64,65,66]. Despite this, including severely ill older adults in clinical trials or interventions, though uncommon, is crucial. This population is the target for developing new methods to support them in managing their illnesses [15,67]. Adopting a flexible design, as we did, can enhance their participation [68].

## 5. Conclusions

Sustaining patient activation is a critical need, and multiple factors may contribute to its enhancement. Our findings suggest that, among older adults with chronic illness, depressive symptoms may act as a barrier to patient activation, indirectly through their association with reduced perceived competence and social relatedness. These findings indicate that interventions targeting competence and relatedness may be valuable to evaluate in future longitudinal or experimental studies. However, the observed associations are consistent with mediation through relatedness and competence, while causal inference remains limited due to the observational nature of the analysis, attrition, and the pooling of randomized controlled trial groups.

## Figures and Tables

**Table 1:** Sample characteristics and demographic data of participants.

	Overall Sample (n = 207)
Men, n (%)	109 (53)
Age	
Mean (SD)	74.8 (10.5)
Md (Q_1_–Q_3_)	76 (68–83)
Have no social support, n (%)	17 (10)
Income (SEK), n (%)	
<10,000	13 (8)
10,001–20,000	75 (46)
20,001–50,000	58 (36)
>50,001	16 (10)
Education, n (%)	
<9 years	11 (6)
Primary school (9 years)	38 (23)
Secondary school	65 (39)
University	51 (32)
Country of birth, n (%)	
Sweden	141 (85)
A Nordic country other than Sweden	11 (7)
Outside the Nordic countries	13 (8)

SD, standard deviation; Md, median; Q_1_–Q_3_, quartile 1 to quartile 3; SEK, Swedish krona.

## Data Availability

The summary data are in the main document. Research data (the dataset with individual data) are not available to share due to both general data protection regulations (GDPR) and limits imposed by the ethics application.

## Abbreviations

The following abbreviations are used in this manuscript:

PAM	Patient Activation Measure
HRQoL	Health-Related Quality of Life
PHQ	Patient Health Questionnaire
NSFS	Need Satisfaction and Frustration Scale
EQ-VAS	EuroQol-Visual Analog Scales
